# Follow-up of implantable cardioverter-defibrillator therapy: comparison of coronary artery disease and dilated cardiomyopathy

**DOI:** 10.1007/s12471-014-0595-z

**Published:** 2014-08-29

**Authors:** M. P. Verhagen, N. van Boven, J. H. Ruiter, G-J. P. Kimman, G. J. Tahapary, V. A. Umans

**Affiliations:** Department of Cardiology, Medical Centre Alkmaar (MCA), Wilhelminalaan 12, 1815 JD Alkmaar, the Netherlands

**Keywords:** Implantable cardioverter-defibrillator, Systolic dysfunction, Coronary artery disease, Dilated cardiomyopathy

## Abstract

**Purpose:**

Since several large trials have proven the effectiveness of implantable cardioverter-defibrillators (ICDs) in patients with left ventricular dysfunction, disadvantages have become more apparent. As the prognosis of patients with cardiovascular diseases is improving, assessment of ICD patients and re-evaluation of the current guidelines is mandatory. We aimed to evaluate differences in mortality and occurrence of (in)appropriate shocks in ICD patients with coronary artery disease (CAD) or dilated cardiomyopathy (DCM).

**Methods:**

In a large teaching hospital, all consecutive patients with systolic dysfunction due to CAD or DCM who received an ICD with and without resynchronisation therapy, were collected in a database.

**Results:**

A total of 320 consecutive patients (age 67 ± 10 years) were classified as CAD patients and 178 (63 ± 11 years) as DCM patients. Median follow-up was 40 months (interquartile range [IQR] 23─57 months). All–cause mortality was 14 % (CAD 15 % vs DCM 13 %). Appropriate shocks occurred in 13 % of all patients (CAD 15 % vs DCM 11 %, *p* = 0.12) and inappropriate shocks occurred in 10 % (CAD 8 % vs DCM 12 %, *p* = 0.27). Multivariate analysis demonstrated impaired left ventricular ejection fraction, QRS >120, age ≥75 years and low estimated glomerular filtration rate as predictors for all-cause mortality. Predictors for inappropriate shocks were permanent and paroxysmal atrial fibrillation.

**Conclusion:**

Mortality rates were similar in patients with CAD and DCM who received an ICD. Furthermore, no differences were found in the occurrence of appropriate and inappropriate ICD interventions between these patient groups.

## Introduction

An implantable cardioverter-defibrillator (ICD) improves survival in patients with impaired left ventricular function [1–4]. Despite these achievements, disadvantages, i.e. inappropriate therapy or non-benefit [5–8], of ICDs have become more apparent over the years and cost-effectiveness should be optimised [9]. As the prognosis of patients suffering from cardiovascular diseases is improving, the current guidelines should be re-evaluated. For this purpose, follow-up data of patients who were assigned to ICD therapy according to the current guidelines are very helpful.

In the current guidelines for device-based therapy and prevention of sudden cardiac death [10], the indications for ICD therapy in patients with an impaired left ventricular function due to dilated cardiomyopathy (DCM) and those for patients with systolic dysfunction due to coronary artery disease (CAD) are quite similar. Therefore, it is of interest to compare the outcome of ICD patients with DCM to ICD patients with CAD, to verify whether the current guidelines are still valid.

The aim of this study was to evaluate mortality and occurrence of both appropriate and inappropriate ICD shocks in patients with an impaired left ventricular function due to CAD and DCM. Furthermore, we assessed predictors for mortality, appropriate and inappropriate shocks.

## Methods

### Study population

A database was constructed including all consecutive patients who received an ICD between January 2005 and June 2012 in a large teaching hospital. Follow-up lasted until October 2012. Therapy assignment was based on the European Society of Cardiology guidelines for device-based therapy [10]. Baseline characteristics of all patients were collected by reviewing hospital records and included demographics, medical history, medication, cardiovascular risk factors and electrocardiographic characteristics. Fifty-two patients who received their ICD for other reasons than CAD or DCM (e.g. idiopathic ventricular fibrillation, hypertrophic cardiomyopathy, arrhythmogenic right ventricular cardiomyopathy, long-QT syndrome, Brugada syndrome, catecholaminergic polymorphic ventricular tachycardia) were excluded. Patients were considered CAD patients if they had a history of myocardial infarction (including Q-wave or enzyme-positive), a history of CAD at coronary angiography or one or more coronary artery bypass grafts or percutaneous coronary interventions. Renal function was assessed by estimating the baseline glomerular filtration rate (eGFR) using the abbreviated Modification of Diet in Renal Disease (MDRD) Study equation: eGFR (mL/min/1.73 m2 of body surface area) =186× (serum creatinine in mg/dL) −1.154 × (age) − 0.203 × 0.742 in female subjects. Renal failure was defined as an eGFR <60 mL/min/1.73 m^2^.

### ICD follow-up

The majority of devices had a three-zone configuration. The first zone was a monitor-only zone, which was set to 160 ± 10 bpm, the VT zone was set to 190 ± 12 bpm and the VF zone was set to >209 ± 15 bpm. In the devices from Medtronic Inc., Minneapolis, MN, USA, the number of intervals to detect was set to 18/24 episodes in all zones. In the devices from Boston Scientific Inc., Indianapolis, IN, USA, the number of intervals to detect was set to 8/10 intervals, with a duration of 8 s in the VT zone and 5 s in the VF zone. For all patients, ICD programming was intended to avoid inappropriate therapy by activating the available discriminators, e.g. dual-chamber algorithms, onset, stability and morphology. For each patient, programming was tailored according to the clinical presentation.

During in-hospital and remote ICD follow-up, as part of usual care, ICD printouts were obtained every 3 months to determine the number and type of arrhythmias and the number of appropriate and inappropriate shocks. Patients were advised to contact the hospital after experiencing ICD therapy or required to visit the hospital if an ICD shock was detected by remote monitoring. ICD therapy was only considered appropriate when delivered for ventricular tachyarrhythmias. All debatable ICD events were double-checked by multiple experts and discussed at a weekly meeting.

### Statistical analysis

Continuous data were analysed with the Student’s t test or Mann–Whitney U test, when appropriate. Categorical characteristics were compared by using the χ^2^ test. Kaplan-Meier’s log-rank test was used to compare differences in all-cause mortality, appropriate shocks and inappropriate shocks between CAD and DCM patients. Furthermore, multivariable Cox proportional hazards regression was used to examine the association between patient characteristics and outcome (hazard ratios, HRs). Characteristics were entered into the multivariable model if they showed a statistically significant association with the outcome during univariable analysis (P value <0.05). Overall statistical significance was set at a 2-tailed P value <0.05. SPSS 20.0 (SPSS Inc, Chicago, IL) was used for the statistical analysis.

## Results

### Study population

The study population consisted of 498 consecutive patients who received an ICD in a large teaching hospital between January 2005 and June 2012. Baseline characteristics are displayed in Table 1. The CAD group comprised 320 patients (64 %). The DCM group consisted of 178 (36 %) patients. The CAD group contained more males than the DCM group (CAD 85 % vs. DCM 62 %, *p* < 0.001) and were older (CAD 67 ± 10 vs DCM 63 ± 11, *p* < 0.047). Mean left ventricular ejection fraction (LVEF) was 24 % ± 7 % and was not significantly lower in one of the groups (25 ± 6 vs 23 ± 8, *p* < 0.131). Permanent atrial fibrillation (AF) was more frequent in DCM patients (8 % vs 18 %, *p* = 0.002).

**Table 1:** Baseline characteristics

Characteristic	All (*n* = 498)	CAD (*n* = 320)	DCM (*n* = 178)	P Value
Age, years	66 ± 10	67 ± 10	63 ± 11	0.047
Male gender	382(77)	272(85)	110(62)	<0.001
LVEF,	24 ± 7	25 ± 6	23 ± 8	0.13
NYHA classification				
I–II	359(80)	233 (81)	126(79)	0.62
III–IV	87 (20)	54(19)	33(21)	
History of atrial fibrillation				
Permanent	59(12)	27(8)	32(18)	0.002
Paroxysmal	52(10)	39(12)	13(7)	0.09
QRS duration, ms	132 ± 32	130 ± 31	136 ± 35	0.003
QRS >120 ms	254(53)	159(52)	95(57)	0.29
Serum creatinine (μmol/L)	117 ± 79	123 ± 90	105 ± 51	0.06
eGFR (ml/min/1.73 m^2^)	65 ± 23	63 ± 23	68 ± 22	0.73
Renal failure	176(40)	121(43)	55(35)	0.13
Haemoglobin (mmol/L)	8.6 ± 1.0	8.6 ± 1.0	8.6 ± 1.1	0.53
Implanted device				
Atrial lead	353(71)	223(70)	130(73)	0.43
CRT	166(33)	89(28)	77(43)	<0.001
Risk factors				
Diabetes	95(19)	69(22)	26(15)	0.06
History of smoking	213(50)	144(53)	69(45)	0.12
Hypertension	147(42)	136(44)	75(44)	0.90
Cardiovascular medication				
Amiodarone	56(11)	42(13)	14(8)	0.08
Beta-blocker	457(93)	294(93)	163(93)	0.95
Digoxin	29(6)	12(4)	17(10)	0.008
ACE inhibitor/ATII antagonist	474(96)	304(96)	170(97)	0.59
Diuretics	382(77)	234(74)	148(84)	0.008

Continuous variables are expressed as mean±standard deviation. Categorical variables are expressed as count (percentage). Valid percentages may vary for some counts, because of missing values. ACE indicates angiotensin-converting enzyme; *ATII* angiotensin-II; *eGFR* estimated glomerular filtration rate; *ICD* Implantable cardioverter-defibrillator; *LVEF* Left ventricular ejection fraction; *NYHA* New York Heart Association

The use of beta-blockers (CAD 93 % vs DCM 93 %, *p* = 0.76) and ACE inhibitors/ARBs (CAD 96 % vs DCM 97 %, *p* = 0.59) did not significantly differ between the groups, but the use of diuretics was higher in patients with DCM (CAD 74 % vs DCM 84 %, *p* = 0.008). A total of 166 patients (33 %) received cardiac resynchronisation therapy (CRT) (CAD 28 % vs DCM 43 %, *p* < 0.001).

### Mortality

**Fig. 1:**
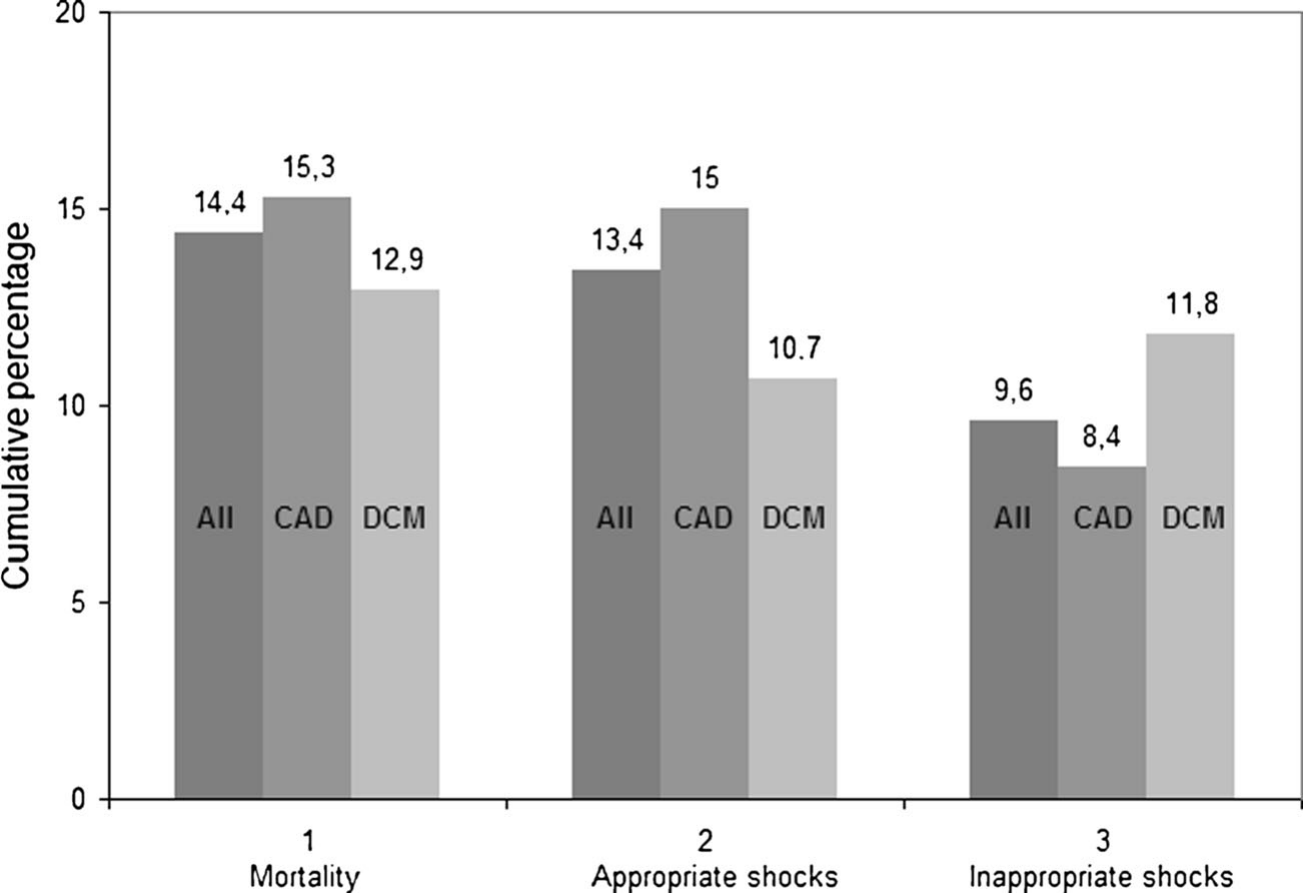
All-cause mortality, appropriate and inappropriate shocks in coronary artery disease (CAD) and dilated cardiomyopathy (DCM) patients

**Fig. 2:**
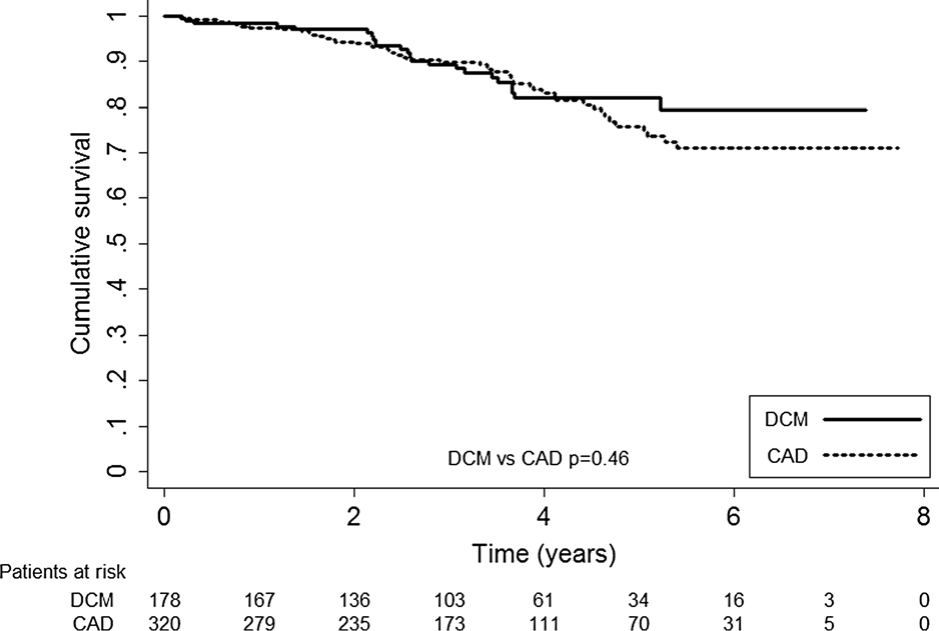
Kaplan-Meier survival curve of all-cause mortality. Coronary artery disease (CAD) versus dilated cardiomyopathy (DCM) patients

Figure 1 displays the results of our outcome on mortality, appropriate and inappropriate shocks. Overall mortality of the total study population was 14.5 % (72 patients), during a median follow-up of 40 months (IQR 23–57 months) with a median survival time of 31 months (IQR 20–44 months). There were no significant differences in mortality between CAD (49 patients, 15 %) and DCM (23 patients, 13 %) patients (*p* = 0.46) (Fig. 2).

Univariable analysis displayed the following predictors for mortality: age ≥75 years, LVEF, New York Heart Association (NYHA) class III-IV, permanent AF, QRS >120 ms, eGFR and haemoglobin. Multivariate analyses showed that impaired LVEF (HR 0.94, CI 0.90–0.99), age ≥75 years (HR 2.18, CI 1.19–3.97), QRS >120 ms (HR 2.50, CI 1.21–5.16) and low eGFR (HR 0.98, CI 0.97–0.99) were independent predictors for mortality.

### Appropriate ICD therapy

**Fig. 3:**
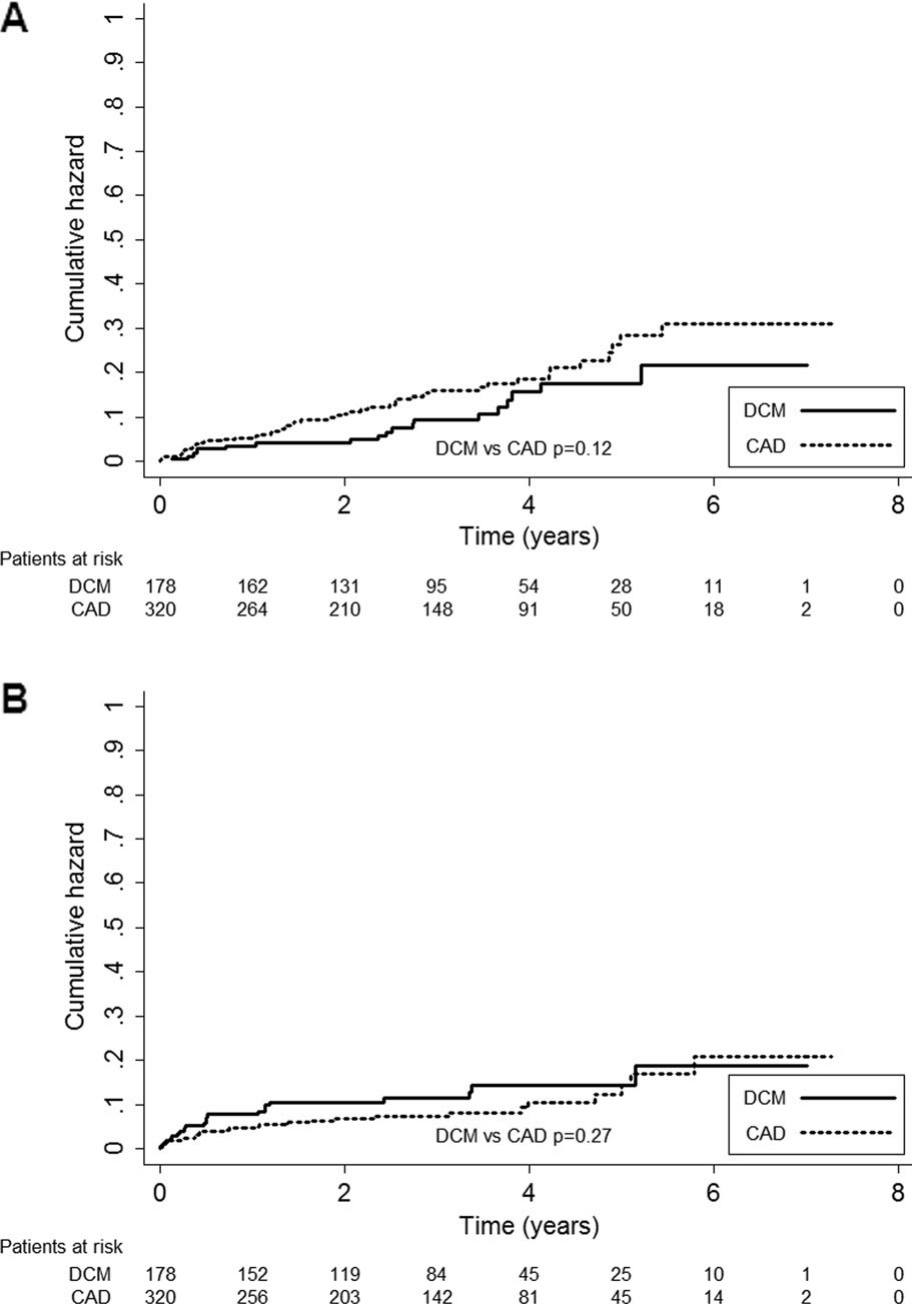
Kaplan-Meier hazard curve of **a** appropriate shocks and **b** inappropriate shocks. Coronary artery disease (CAD) versus dilated cardiomyopathy (DCM) patients

A total of 67 patients (13.5 %) received ≥1 appropriate shocks and 43 patients (9 %) received >1 appropriate shocks during follow-up. The median interval to first appropriate shock after ICD implantation was 21.8 months (IQR 4.9–35.0 months). Cumulative incidence of appropriate shocks was 4.4, 7.2 and 13.1 % at 1, 2 and 5 years follow-up, respectively. There were no significant differences in the occurrence of appropriate shocks between CAD patients and DCM patients (CAD 15.0 % vs DCM 10.7 %, *p* = 0.12) (Fig. 3a). Use of digoxin (15 vs 4 %, HR 2.97, CI 1.50–5.88) and a history of smoking (62 % vs 48 %, HR 2.00, CI 1.77–2.98) predicted appropriate shocks.

### Inappropriate ICD therapy

Inappropriate ICD shocks occurred in 48 patients (9.6 %). Twenty-four patients (5 %) had >1 episode of inappropriate shocks. The median time between implantation and the first inappropriate shock was 6.1 months (IQR 2.3–26.6 months). Cumulative incidence of inappropriate shock therapy was 5.4, 7.2 and 9.0 % at 1, 2 and 5 years of follow-up. There was no significant difference in occurrence of inappropriate shock therapy between CAD patients and DCM patients (CAD 8 % vs DCM 12 %, *p* = 0.27) (Fig. 3b). Inappropriate shocks occurred significantly more in patients with permanent AF (22 vs 8 %, p = 0.001), and also in patients with paroxysmal AF (17 vs 9 %, *p* = 0.048). Most inappropriate shocks in this study were caused by supraventricular tachyarrhythmias (78 %), mainly AF. Other causes of inappropriate shocks were shock lead dysfunction (18 %) and T wave oversensing (4 %). Multivariate analyses displayed permanent AF (HR 2.85, CI 1.16-7.01) and paroxysmal AF (HR 2.84, CI 1.20–6.74) as independent predictors for inappropriate shocks.

## Discussion

We performed a retrospective, observational, follow-up study, on 498 real-life patients, treated with an ICD and evaluated the difference in mortality and occurrence of ICD shocks in patients with left ventricular dysfunction due to CAD versus DCM. All patients received their ICD according to the current guidelines.

The major findings of this study were: (1) Mortality rates are equal in CAD and DCM patients; (2) Incidence of appropriate and inappropriate shocks was similar in both groups; (3) Predictors for mortality in ICD patients were impaired LVEF, age ≥75 years, QRS >120 ms and low eGFR; (4) Predictors for inappropriate ICD intervention were permanent and paroxysmal AF.

### Mortality

Overall cumulative incidence of all-cause mortality of the total study population was 14.5 % and at 1, 2 and 5 years, mortality rates were 2.2, 4.2 and 13.5 %, respectively. For CAD patients, mortality rates at 1, 2 and 5 years were 2.5, 5.3 and 14.1 % respectively, and for DCM patients 1.7, 2.8 and 12.4 %. Compared with the landmark trials, the cumulative incidence off all-cause mortality was relatively low. The Sudden Cardiac Death in Heart Failure Trial (SCD-HeFT) found a total mortality rate of 29 % at 5-year follow-up and the Multicenter Automatic Defibrillator Implantation Trial II (MADIT-II) reported a mortality rate of 16 % at 2 years follow-up and 52 % at 8 years [2, 3, 11]. This lower mortality could be explained by the fact that treatment of patients with systolic dysfunction has improved over time, which may have contributed to a lower mortality in our study population compared with the trials mentioned. Another factor contributing to the lower mortality could be due to the fact that 33 % of our study population received CRT, which improves left ventricular function and reduces mortality [12].

As ICD implantation and follow-up is expensive and as costs in medical practice are rising, a stricter selection of eligible patients is mandatory. The relatively low mortality in our study population calls for reassessment of the indications for an ICD. Therefore, further studies and registries of real-life ICD patients are required to make a more appropriate selection of patients eligible for ICD implantation possible.

Finally, whereas ICDs only act as a ‘safety net’ and antiarrhythmic medication has potential side effects and requires close monitoring, the search for other methods to withstand arrhythmias continues. Catheter ablation is an accepted technique and nowadays commonly used in the treatment of arrhythmias. Catheter ablation has proven its effectiveness in the treatment of VTs in patients with structural heart disease due to CAD or DCM with even higher success rates in CAD patients [13].

### Appropriate intervention

In our study, 13.5 % of all patients received appropriate shocks, and this number did not significantly differ between CAD and DCM patients. The SCD-HeFT trial reported a total number of appropriate shocks of 21 %, which is higher than the 13 % of all patients receiving appropriate shocks that we reported. This difference might be clarified by the fact that device programming has improved over time and antiarrhythmic medication has been enhanced. Also, as stated before, 33 % of our study population received CRT, which could also have had a beneficial effect on the number of appropriate shocks by increasing LVEF.

There is some evidence that the number of ventricular tachyarrhythmias is comparable in CAD patients and DCM patients.[14, 15] This had also been shown by some small previous studies.[16, 17] Our study adds to these findings by showing that the number of ICD shocks is also equivalent in these two groups, even though one-third of our patients received CRT, which may have a more beneficial effect on LVEF in DCM patients compared with CAD patients.[18] Our findings confirm the validity of the current guidelines.

Recently, studies have shown that medication indeed reduces appropriate ICD therapy in patients with ischaemic heart disease [19–21]. Since ventricular tachyarrhythmias are the major cause of sudden cardiac death, it is important to reduce these arrhythmias and therefore appropriate shocks, which have also proven to be an independent predictor of mortality [22].

Our study reveals that a history of smoking is a predictor for appropriate shocks, which has been shown before in previous studies [23]. Smokers have increased atherosclerosis, which increases the occurrence of ischaemic events, and myocardial scarring, eventually resulting in more tachyarrhythmias and consequently more appropriate shocks.

Finally, most of the patients who received an ICD never received shock therapy, suggesting that a more patient-focused risk stratification could improve clinical benefits and cost-effectiveness [24].

### Inappropriate intervention

In this study, inappropriate shocks occurred in 9.6 % of all patients, mostly caused by atrial tachyarrhythmias classified as ventricular tachyarrhythmias, which subsequently caused inappropriate discharges.

Age <75 years was also associated with inappropriate shocks. Younger age is associated with sinus tachycardia and abnormal sensing. This finding has been reported before [25], and is most likely the explanation for the association between age <75 years and inappropriate shocks. Patients who had an ICD as secondary prevention had a slightly better LVEF compared with the primary prevention patients and a lower NYHA class. Possibly, these patients were more physically active, which could also lead to a higher number of shocks from sinus tachycardia or abnormal sensing.

The most frequent causes of inappropriate therapy have been studied, and can result in reduced quality of life and even provocation of ventricular arrhythmias.[5, 26] An additional phenomenon is phantom shocks - the sensation of an ICD discharge in the absence of an actual discharge – which occur, though not significantly, more in patients who received appropriate or inappropriate shocks [27]. Therefore, the occurrence of inappropriate shocks should be minimised as much as possible. Recent studies have shown that enhanced programming algorithms during follow-up reduces inappropriate therapy and even mortality [28, 29].

### Limitations

The present study has several limitations. Patients were included in the period between January 2005 and June 2012, so follow-up of the last included patients was only 3 months while some patients have a follow-up of up to 7 years. Within this period of time, multiple publications on treatment of ventricular tachyarrhythmias and device-based therapy have changed the selection of eligible patients for ICD treatment. This could have caused heterogeneity in the study population, which may have affected the outcome. This limitation did not influence the aim of the study, since this limitation applies to both CAD and DCM patients.

A second limitation is the fact that this study was performed retrospectively, which makes data collection challenging. Nevertheless, all data on primary and secondary outcomes could be collected without loss to follow-up.

### Conclusion

This study shows that mortality and occurrence of appropriate and inappropriate ICD shocks are similar in patients with an ischaemic or a dilated cardiomyopathy. An impaired LVEF, age ≥75 years, QRS >120 ms and low eGFR predicted mortality. Use of digoxin and a history of smoking predicted appropriate shocks. Permanent AF and paroxysmal AF are predictors for inappropriate shocks.
